# Renal autoregulation and blood pressure management in circulatory shock

**DOI:** 10.1186/s13054-018-1962-8

**Published:** 2018-03-22

**Authors:** Emiel Hendrik Post, Jean-Louis Vincent

**Affiliations:** Department of Intensive Care, Erasme Hospital, Université Libre de Bruxelles, Route de Lennik 808, 1070 Brussels, Belgium

**Keywords:** Acute kidney injury, Cardiogenic shock, Hemorrhagic shock, Renal blood flow, Septic shock

## Abstract

The importance of personalized blood pressure management is well recognized. Because renal pressure–flow relationships may vary among patients, understanding how renal autoregulation may influence blood pressure control is essential. However, much remains uncertain regarding the determinants of renal autoregulation in circulatory shock, including the influence of comorbidities and the effects of vasopressor treatment. We review published studies on renal autoregulation relevant to the management of acutely ill patients with shock. We delineate the main signaling pathways of renal autoregulation, discuss how it can be assessed, and describe the renal autoregulatory alterations associated with chronic disease and with shock.

## Background

A number of clinical studies have reported an association between low arterial blood pressure and increased mortality in patients with different types of shock [1, 2]. These patients typically receive vasopressor therapy to maintain adequate organ perfusion [3]. Generally, a mean arterial pressure (MAP) target of around 65 mmHg is recommended in the initial management of shock, but the optimal level is hard to define [4, 5]. For example, a lower target may be advisable in patients with hemorrhagic shock without severe brain injury to minimize additional blood loss [6]. Conversely, some patients may benefit from a higher blood pressure than others. Indeed, a small study in 25 patients with cardiogenic shock suggested a beneficial effect on microcirculation and tissue metabolism of increasing MAP from 65 to 85 mmHg [7]. Whether a higher blood pressure beneficially affects renal blood flow (RBF) or kidney function is unknown. In septic shock, variable responses of RBF and kidney function to blood pressure levels greater than 65 mmHg have been reported in several small interventional studies [8–10]. The randomized controlled SEPSISPAM trial evaluated the effect of increasing the target MAP to 80–85 mmHg, compared to a target of 70–75 mmHg, in 776 septic shock patients and found no difference in mortality between the two groups [11]. However, patients in the pre-defined subgroup with arterial hypertension benefited from the higher MAP, as evidenced by lower plasma creatinine concentrations and reduced use of renal replacement therapy. These findings may be attributed to the existence of impaired renal autoregulation in these patients.

## Mechanisms of renal autoregulation and its assessment

**Fig. 1:**
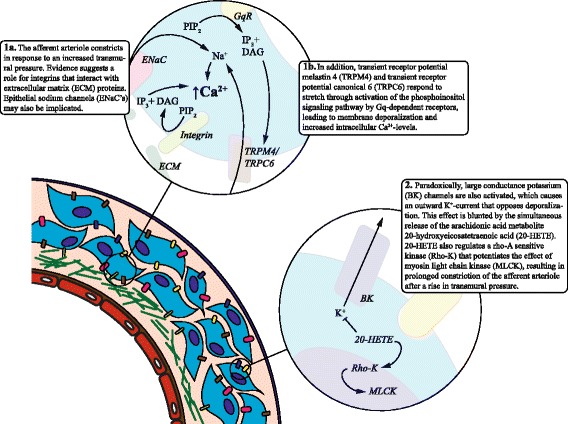
Main elements of the signaling pathway underlying the myogenic response

**Fig. 2:**
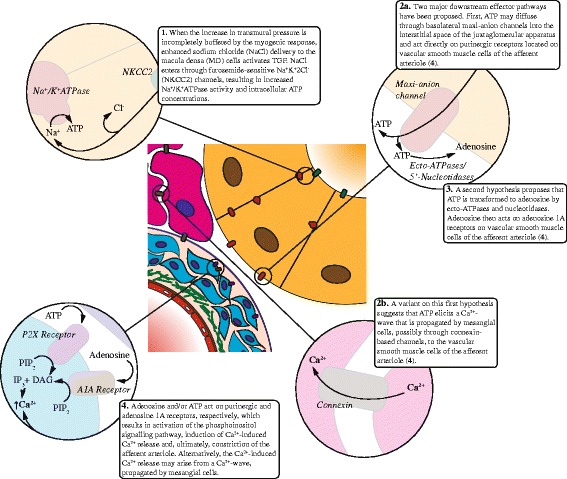
Main elements of the signaling pathway underlying tubuloglomerular feedback (TGF)

At least two different mechanisms contribute to renal autoregulation: the fast, myogenic, and the slower, tubuloglomerular feedback (TGF), responses. Figures 1 and 2 illustrate the main events in the signaling cascades of the myogenic and TGF responses, respectively. Burke et al. [12] have reviewed the molecular mechanisms of renal autoregulation in more detail. The experimental manipulation and data transformation necessary to investigate renal autoregulation can be a source of confusion. The following paragraphs provide a general outline of this subject. A more exhaustive overview can be found elsewhere [13, 14].

**Table 1:** Characteristics of studies that have used animal models relevant to the critically ill patient population

Study	Species	Model	Interventions	Key findings
Adams et al. 1980 [46]	Dog	Ischemia/reperfusion (clamp time 90 min)	-	Diminished autoregulatory efficiency at 18 h
Matthys et al. 1983 [47]	Rat	Ischemia/reperfusion (clamp time 45 min)	-	Loss of autoregulation at 48 h and 7 days after clamping
Williams et al. 1981 [48]	Dog	Ischemia/reperfusion (clamp time 60–90 min)	-	Attenuated autoregulatory reserve at 18 to 24 h after clamping. Strong negative correlation autoregulatory index and inulin clearance
Verbeke et al. 1998 [49]	Rat	Ischemia/reperfusion (clamp time 40 min)	Ketanserin	Renal autoregulation lost at 2 and 24 h after clamping. Ketanserin partially restored autoregulation at 24 but not at 2 h
Conger et al. 1991 [50]	Rat	Ischemia/reperfusion by clamping or NE-infusion (clamp time 75 min; NE at 0.6 μg/kg/min for 90 min)	-	Impaired autoregulatory efficiency at 1 week, worse with NE vs clamping
Conger et al. 1994 [51]	Rat	4-h MAP reduction by phlebotomy, 1 week after ischemia/reperfusion by clamping or NE-infusion (clamp time 75 min; NE at 0.6 μg/kg/min for 90 min)	-	Impaired autoregulatory efficiency, worse with clamping vs NE, associated with functional and structural deficits
Guan et al. 2006 [52]	Rat	Ischemia/reperfusion by unilateral or bilateral clamping for 30 or 60 min	-	
Rhee et al. 2012 [32]	Piglet	Hemorrhagic shock; stepwise MAP reductions from 80 to 60, 45, and 40 mmHg	-	Early passive pressure–flow relationship between blood pressure and RBF suggests loss of renal before cerebral autoregulation
Burban et al. 2013 [57]	Rat	Cecal ligation and puncture. Reconstruction of autoregulatory curve using MAP reductions by acute bleeding	-	Renal autoregulation unaffected by sepsis, with or without NE
Nitescu et al. 2010 [58]	Rat	Endotoxemia by LPS bolus infusion. Dynamic analysis of renal autoregulation	Candesartan	Reduced transfer gain values in TGF-associated frequency range after 16 h, which normalized with angiotensin II receptor antagonism

*NE* norepinephrine, *LPS* lipopolysaccharide, *MAP* mean arterial pressure, *TGF* tubuloglomerular feedback, *RBF* renal blood flow

### Static renal autoregulation

**Fig. 3:**
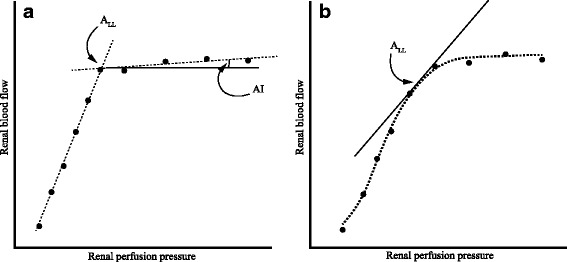
Static renal autoregulation. **a** Linear regression can be used to fit two straight lines to the data. **b** If there is a more gradual transition to pressure-dependent flow, a logistic curve may be fitted. The shoulder of the curve can be calculated and used to define the lower limit of renal autoregulation (A_LL_). *AI* autoregulatory index

Renal autoregulation is often characterized by measuring the steady-state response of whole-organ RBF to adjustments in renal perfusion pressure (RPP). In rodent models, a decrease in RPP is usually achieved by inflating a cuff placed around the abdominal aorta, immediately above the renal arteries, whereas the anatomy of large animals allows the cuff to be placed around the renal artery itself. To evaluate autoregulatory behavior at higher RPP ranges, bilateral carotid artery occlusion is applied or vasopressors are administered. It is important to note that sympathetic discharge and vasopressor agents can directly affect renal autoregulation and may, therefore, complicate interpretation of the results [15]. The renal autoregulatory relationship is reconstructed by fitting an appropriate curve to the data. Classically, linear regression is used to fit two straight lines, one to the data points below the lower threshold, where RBF is pressure-dependent, and another to the data points located on the autoregulatory plateau (Fig. 3a). The lower limit of renal autoregulation (A_LL_) is defined as the pressure level that corresponds to the intersection of these lines, and the steepness of the plateau defines the autoregulatory efficiency, or autoregulatory index (AI) [16]. When the transition from autoregulated to pressure-dependent flow is less clear, a smoother, e.g., logistic, curve can be fitted [17] (Fig. 3b).

### Dynamic renal autoregulation in the time domain

**Fig. 4:**
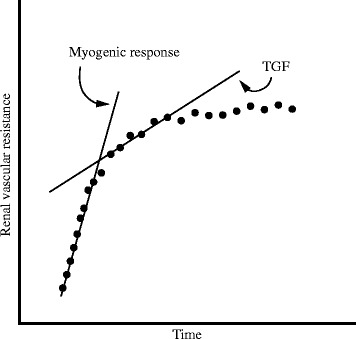
Dynamic renal autoregulation in the time domain: the myogenic response generates the initial, steep rise in renal vascular resistance. Tubuloglomerular feedback (*TGF*) starts to contribute after approximately 5–10 s

Adapting renal vascular resistance to changes in RPP is a dynamic process, and the renal vascular bed has a certain response time that can be measured to further define the system. This characterization is appealing as these response times vary according to the different mechanisms underlying renal autoregulation [18]. Performing the appropriate experimental manipulation thus enables the investigator to estimate the separate contributions of each of these mechanisms to the overall response (Fig. 4). This manipulation usually consists of applying an acute increase in RPP and monitoring the ensuing change in renal vascular resistance (RVR) over time. The myogenic mechanism responds within seconds, resulting in an initial steep increase in the RVR curve [19]. The curve’s steepness typically wanes after about 10 s, which corresponds to the moment when TGF starts to contribute to the autoregulation [20]. Although this method allows the evaluation of each mechanism’s response time and its contribution to the complete response, it does not provide a measure of total autoregulatory efficiency. Moreover, it only evaluates the autoregulatory response to an increase, not a decrease, in RPP. Translating the data to the frequency domain can partially resolve this limitation.

### Dynamic renal autoregulation in the frequency domain

**Fig. 5:**
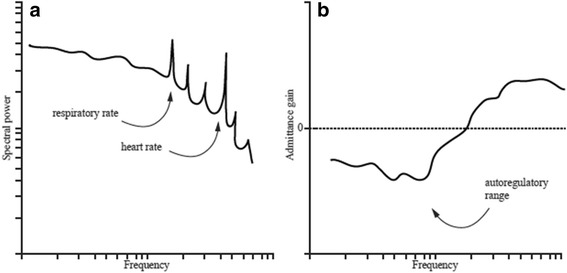
Dynamic renal autoregulation in the frequency domain. **a** Power spectra for pressure and flow are constructed from their respective time series. **b** Both power spectra are combined to construct a transfer gain function. Negative gain values imply effective renal autoregulation in the given frequency range

Blood pressure signals contain spontaneous oscillations at varying frequencies, notably at those corresponding to the heart and respiratory rates, but also at lower frequencies that likely arise from oscillations in sympathetic vasomotor activity [21]. These pressure oscillations can be visualized in a power spectrum after the time series has been mathematically translated into the frequency domain, usually by fast Fourier transformation (FFT; Fig. 5a). When a similar transformation is applied to the flow signal, both spectra can be combined to construct a transfer gain function (Fig. 5b). A low transfer gain value implies that oscillations in blood pressure do not translate into flow fluctuations of similar magnitude, i.e., the kidney is effectively autoregulating in the given frequency range. As for the time domain, this analysis allows identification of the separate components: the myogenic response is thought to operate between 0.1 and 0.2 Hz, whereas TGF typically dampens oscillations at frequencies below this range [22]. This analysis thus allows quantification of autoregulatory efficiency and identification of the underlying mechanisms, typically without active manipulation of RPP. However, there are still two important limitations: first, FFT assumes linearity between the input and output signal, but the renal autoregulatory system usually displays at least some degree of non-linear behavior [23]. Second, FFT assumes the properties of the system are stationary or have constant mean and variance over time; however, this assumption may not always be valid, particularly when prolonged time series are used in hemodynamically unstable subjects [24].

### Dynamic renal autoregulation: nonlinear and time-varying models

Because of the limitations mentioned in the last paragraph, attention is being focused on the development of nonlinear methods with higher temporal resolution [25]. Discussion of the details of these models is beyond the scope of this review, but these new methods hold promise for the investigation of renal autoregulatory features in the presence of relatively unstable hemodynamics, as in some acute illnesses.

### Assessment of renal autoregulation in clinical practice

The clinical evaluation of renal autoregulation is restricted because of the limitations of non-invasive RBF assessment. For example, renal Doppler-derived indices, such as those applied by Lerolle et al. to evaluate the association between renal vascular resistance and renal function in patients with septic shock [26], may correlate poorly with actual changes in renal hemodynamics [27]. Moreover, displacement of the kidney during the respiratory cycle limits the use of renal Doppler in the evaluation of renal dynamic autoregulation; whereas cerebral Doppler has been successfully used to assess cerebral autoregulation in human volunteers injected with endotoxin [28]. Contrast-enhanced ultrasound may be an alternative means to evaluate RBF in critically ill patients [29]. In this technique, dedicated microbubbles are destroyed using high-power ultrasound pulses, and the time needed for replenishment is used as a marker of RBF [30]. Similarly, phase-contrast magnetic resonance imaging (MRI) has been successfully used to estimate RBF in critically ill patients [31]. Both techniques, however, measure RBF over a relatively short time period, which prevents their use in the evaluation of renal dynamic autoregulation. Rhee et al. [32] successfully evaluated renal autoregulation in piglets with hemorrhagic shock (see below) using near-infrared spectroscopy (NIRS). Although this technique allows for continuous, non-invasive monitoring of organ blood volume, its low penetration depth limits its application to the pediatric population [33]. Finally, Redfors et al. [9] evaluated the RBF response to graded norepinephrine infusions using a renal vein catheter and continuous retrograde thermodilution in patients with vasodilatory shock. It therefore seems that the evaluation of renal autoregulation in human subjects may be feasible, but is technically challenging and knowledge on renal autoregulation is therefore mostly derived from studies in animal models.

## Renal autoregulation and the influence of comorbidities

The primary function of renal autoregulation in physiological circumstances is to prevent excessively high systolic blood pressures from reaching, and damaging, the glomerular vascular structure [34]. An important body of evidence connects chronic arterial hypertension to alterations in renal autoregulatory properties [35]. Indeed, the rightward shift of the autoregulation curve that occurs with chronic hypertension likely serves to maintain this protective effect at higher default pressures. For example, in animal models of hypertension, subjects that do not exhibit this adaptive response appear to be at an increased risk of developing chronic kidney disease [36]. Clinical data are in line with this hypothesis, as a shifted A_LL_ was demonstrated in patients with severe, but not moderate, hypertension [37]. Kotchen et al. [38] observed impaired autoregulation of glomerular filtration rate (GFR), but not RBF, in African-American patients with moderate hypertension. The use of calcium channel blockers, known to impair the myogenic response, resulted in a lower GFR in a similar population [39]. Textor et al. [40] found that generalized atherosclerosis, in the absence of systemic hypertension, was also associated with impaired renal autoregulation. The presence of other chronic diseases, particularly type 2 diabetes mellitus, may also affect renal autoregulatory properties. Renal autoregulation was impaired in most [41], but not all, rat models of type 2 diabetes mellitus [42]. A series of clinical studies by Christensen et al. [43–45] described the presence of impaired renal autoregulation in human type 2 diabetes mellitus.

## Renal autoregulation in critical illness

### Renal autoregulation in hemorrhagic and cardiogenic shock

Adams et al. [46] were the first to study renal autoregulation in a model of canine renal ischemia and reperfusion (Table 1). They observed profoundly impaired static autoregulatory efficiency at 18 h after a 90-min period of renal ischemia. These findings have been reproduced following ischemic injury in different species [47], after shorter ischemic times [48], and after shorter [49] and longer [50, 51] observation times. Guan et al. [52] suggest that increased availability of nitric oxide (NO), derived from endothelial- but not inducible-NO synthase (NOS), may be involved in the loss of renal autoregulation following renal ischemia.

Stopping RBF completely does not, however, adequately represent the clinical scenario of cardiogenic or hemorrhagic shock and more relevant models need to be considered. In a piglet model of hemorrhagic shock, Rhee et al. [32] investigated renal autoregulation by calculating a moving correlation coefficient between slow changes in RPP and laser Doppler- or near-infrared spectroscopy-derived flow values. There was an early increase in correlation coefficient in the kidney compared to the brain, implying a passive renal pressure–flow relationship early in the course of hemorrhagic shock. Indeed, this feature enables rapid reduction in RBF in case of hypovolemia, retaining circulating blood volume and diverting it towards more vital organs [53].

Indirect evidence provides some support for this observation. For example, increased renal sympathetic nerve activity (RSNA), typically present in hemorrhagic shock [54], was implicated in the development of a high AI, or impaired autoregulatory efficiency, in a rat model of acute kidney injury induced by norepinephrine infusion [55]. Similarly, increasing RSNA by carotid occlusion shifted the A_LL_ to the right by more than 25 mmHg in healthy dogs [15], whereas renal denervation reduced transfer gain values at frequencies < 0.01 Hz in hypertensive rats with pathologically high levels of RSNA [56]. Furthermore, a frequency analysis study in healthy rats showed that the presence of a low MAP, albeit still within the autoregulatory range, negatively affected the gain reduction in the renal myogenic mechanism [22].

### Renal autoregulation in septic shock

Burban et al. [57] investigated renal autoregulation in a rodent model of abdominal sepsis by bleeding the animals to reduce RPP. Early sepsis did not seem to affect the relationship between RBF and arterial blood pressure, although A_LL_ and AI were not quantified and the considerable blood loss made this essentially a two-hit model [57]. Nitescu et al. [58] applied frequency analysis in rats injected with lipopolysaccharide and concluded that TGF, but not the myogenic response, was negatively affected by endotoxemia. The extent to which these findings represent clinical sepsis, however, is unclear, as the animals still had a mean MAP of 120 mmHg after 16 h of endotoxemia.

As in hemorrhagic shock, circumstantial evidence suggests that renal autoregulation may be affected in septic shock. Most importantly, NO, produced in large quantities during sepsis [59], is known to modulate renal autoregulation. Studies have revealed that non-specific inhibition of NOS decreased transfer gain values in the myogenic- [60] and TGF-associated frequency ranges [61]. Moreover, NOS inhibition augmented the myogenic response to both a step increase and decrease in RPP when TGF was inhibited by furosemide [20]. This observation suggests an important interaction between the two mechanisms [62], which may also explain why NOS inhibition does not seem to affect static autoregulatory indices [63–65]. Mitrou et al. [66] showed that NO may also impair the synchronization of renal autoregulation that normally occurs between cortical nephrons from the same lobule.

Other known mediators of septic renal microcirculatory dysfunction include reactive oxygen species (ROS) and endothelin-1. Studies have yielded conflicting results, with ROS shown to enhance the myogenic response [67] and regulate TGF [68], but also to abolish autoregulation in juxtamedullary afferent arterioles [69]. Conversely, renal hypoxanthine/xanthine oxidase-generated superoxide production in rats did not affect autoregulation of RBF [70]. Likewise, endothelin type A receptor antagonism did not alter renal autoregulatory efficiency in dogs [71], whereas endothelin type B receptor blockade enhanced the myogenic response in healthy rats [72].

### The effects of vasopressors and fluid therapy on renal autoregulation

Only a few vasopressors have been studied in the context of renal autoregulation. As already described above, NOS inhibitors improve renal autoregulatory efficiency independently from their effects on RPP. In healthy dogs, Ogawa and Ono compared norepinephrine with angiotensin II during infusion of the L-type calcium channel blocker, verapamil, which effectively blocks the myogenic response, and found that neither molecule influenced the impaired autoregulatory relationship [73]. Angiotensin II seems to have only a minor influence on renal autoregulation in healthy animals [74], but may be essential to reset the A_LL_ in cases of systemic hypotension [75]. Kiil et al. [76] compared static autoregulatory efficiency during angiotensin II and norepinephrine infusions in anesthetized healthy dogs, and noted that norepinephrine shifted the A_LL_ to the right, i.e., it decreased the range of renal autoregulatory activity compared to angiotensin II and vehicle infusion. Finally, Wang et al. investigated the effects of arginine vasopressin on transfer gain values in normotensive rats, and reported a minor increase in gain values with vasopressin in the lower frequency range, although the persistently negative gain values imply that the kidney was still autoregulating effectively [77].

No studies have been done on the effects of fluid therapy on renal autoregulation. However, since hematocrit levels affect vascular wall shear stress and NO release, thus modulating peripheral vascular resistance [78, 79], it is possible that fluid administration may influence renal autoregulatory efficiency. Indeed, a small observational study in healthy volunteers showed that central hypervolemia and hemodilution were associated with impaired cerebral autoregulation [80].

## Remaining questions and future research

Studies have revealed that low MAP may be associated with increased mortality in cardiogenic shock [1, 2]. Similarly, multiple observational studies in septic shock have reported that repetitive reductions in MAP or RPP are associated with adverse renal outcome [81]. So far, this has mainly raised discussion about which MAP threshold should be targeted when titrating norepinephrine in these patients. Indeed, results from the SEPSISPAM study [11] suggest that septic patients with previous arterial hypertension may benefit from higher MAP targets, which is in line with the rightward shift of the A_LL_ observed in many experimental models of hypertension. However, some data suggest that not all patients with hypertension display a similar shift. For example, patients with severe hypertension and signs of renal injury are likely to have no renal autoregulation and whether aiming for a higher RPP would be beneficial in these cases, particularly in view of the protective properties of renal autoregulation, remains unknown. Furthermore, experimental data indicate that the choice of vasopressor could have a direct influence on renal autoregulation, which may help to reduce the risk of renal hypoperfusion in shock. Finally, whether fluid administration affects renal autoregulation is unknown.

## Conclusions

The recent focus on the role of renal autoregulation seems justified but more experimental and clinical studies are needed to provide high quality evidence that could help us to better guide blood pressure management in critically ill patients.

## Abbreviations

AI: Autoregulatory index
A_LL_: Lower limit of autoregulation
FFT: Fast Fourier transformation
GFR: Glomerular filtration rate
MAP: Mean arterial pressure
NO: Nitric oxide
NOS: Nitric oxide synthase
RBF: Renal blood flow
ROS: Reactive oxygen species
RPP: Renal perfusion pressure
RSNA: Renal sympathetic nerve activity
RVR: Renal vascular resistance
TGF: Tubuloglomerular feedback
